# MUC15 is an independent prognostic factor that promotes metastases of MYCN non-amplified neuroblastoma

**DOI:** 10.7150/jca.89360

**Published:** 2023-10-16

**Authors:** Huiqin Guo, Wei-Xin Zhang, Qiu-yan Zhang, Meng Li, Hai-Yun Wang, Di Li, Jiabin Liu, Zhenjian Zhuo, Jing He, Lei Miao, Huimin Xia

**Affiliations:** 1 School of Medicine, South China University of Technology, Guangzhou, Guangdong, China.; 2 Department of Pediatric Surgery, Guangzhou Institute of Pediatrics, Guangdong Provincial Key Laboratory of Research in Structural Birth Defect Disease, Guangzhou Women and Children's Medical Center, Guangzhou Medical University, Guangzhou 510623, Guangdong, China.; 3 Department of Pharmacology, School of Pharmacy, Binzhou Medical University, Yantai, 264003, China.; 4 Laboratory Animal Center, School of Chemical Biology and Biotechnology, Peking University Shenzhen Graduate School, Shenzhen 518055, China.

**Keywords:** MUC15, neuroblastoma, MYCN non-amplified, metastases, MYCT1

## Abstract

**Background:** Neuroblastoma (NB) is a cancer that arises from neural-crest-derived sympathoadrenal lineage. Less is known about the pathogenesis and molecular characteristics of MYCN non-amplified (MYCN-NA) NB.

**Methods:** We constructed a signature model targeting mucin family according to RNA sequencing data from GSE49710 dataset, and validated the prognostic performance. We also analyzed the gene expression matrix using DESeq2 R packages to screen the most differential mucin in high-risk NB samples. We further assessed its prognostic value, particularly in MYCN-NA NB samples. Moreover, we performed functional experiments to evaluate the impact of MUC15 overexpression on the migration of MYCN-NA NB cell lines.

**Results:** The 8-mucin signature model showed good prognostic performance in the GSE49710 dataset. Among the mucin genes, MUC15 was significantly upregulated in the high-risk NB cohort and was associated with poor prognosis, especially in MYCN-NA NB samples. Furthermore, MUC15 overexpression and exogenous MUC15 protein enhanced the migration of MYCN-NA NB cell lines. Mechanistically, MUC15 promoted the phosphorylation of focal adhesion kinase (FAK) by inhibiting the expression of MYCT1, a target of c-Myc.

**Conclusions:** Our findings suggested a potential network in controlling NB cell metastasis. Targeting MUC15 in MYCN-NA NB patients could be a promising therapeutic strategy.

## 1. Introduction

NB is the typical extracranial solid tumor that derives from sympathetic nervous system among childhood and represents roughly 15% in pediatric malignant tumor mortality 1. The adrenal gland, abdomen and thorax are the frequent regions for NB 2. Risk parameters have been established to determine the prognosis and treatment options for NB patients. Those with very low, low, and intermediate risks have a more favorable prognosis and treatment outcomes 3. However, over 60% of NB patients are confirmed as high risk. Despite undergoing integrated treatment including radiotherapy, chemotherapy and immunotherapy, the 5-year event-free survival rate (EFS) of high-risk patients is still about 50% 4. The heterogeneity of high-risk NB leads to poor outcomes with frequent recurrence and metastasis. Apart from age, the status of MYCN gene amplification is a principal factor in determining the risk grading. The EFS for NB patients with MYCN-amplified (MYCN-A) and MYCN-NA is 51.1% and 77.0% respectively 4. Although many genetic abnormalities and molecular mechanisms have been discovered, the prospect of high-risk patients is still not optimistic 1, 5. Compared to MYCN-A tumors, less is known about the pathogenesis and molecular characteristics of MYCN-NA NB. Therefore, there is an urgent need for molecular therapies targeting the MYCN-NA subgroup to address the treatment dilemma.

Mucins (MUCs) are highly glycosylated membrane proteins expressed broadly in epithelial cells. They have been characterized as transmembrane and secretory isoforms, which serve as protective barriers and transmit signals to the cell interior. Secretory mucins include MUC2, MUC5AC, MUC5B, MUC6 and MUC19, while transmembrane mucins include MUC1, MUC3, MUC4, MUC12, MUC13, MUC15, MUC16, MUC17, MUC21 and MUC22 6, 7. MUC1 and MUC16 have been broadly studied for their extensively aberrations in malignancy 8-13. MUC1, also known as CA153 or CA199 in clinical diagnosis, interacts with various transcription factors such as p53, STAT3, NF-κB and β-catenin. These interactions activate multiple signaling pathways, including mitogen-activated protein kinase (MAPK), phosphatidylinositol 3-kinase (PI3K)/AKT and wingless type (Wnt), which contribute to malignant transformation in tumors 14. On the other hand, MUC16, also refers to CA125, is highly expressed in breast, ovarian and pancreatic cancer. It is clinically used as a tumor marker for ovarian cancer 15. MUC16 interacts with Janus kinase (JAK2) to induce STAT3 phosphorylation, thereby promoting the proliferation of breast and lung cancer cells 11, 12. Both MUC1 and MUC16 play a role in mediating the epithelial-mesenchymal transition (EMT) process of tumor cells and facilitating metastasis 13. Aberrant glycation in mucins is associated with tumor immune evasion and signal transduction issues, making them potential markers of tumor prognosis and treatment response 16.

In our study, we identified an 8-mucin signature model that showed good prognostic value in the GSE49710 dataset. Specifically, MUC15 was found to be upregulated in the high-risk cohort, particularly in MYCN-NA NB samples. MUC15 belongs to the family of transmembrane mucins. Its molecular structure consists of an N-terminal region and a C-terminal region. The N-terminal region contains a signal peptide and an extracellular domain formed by 10 N-glycosylated and 14 O-glycosylated modifications. The C-terminal region includes a transmembrane domain and a cytoplasmic tail 17. Abnormal expression of MUC15 plays a crucial role in malignant tumors, affecting cell growth, adhesion, invasion and metastasis. However, the role of MUC15 in cancer is context-dependent and even opposite among different tumors. In some cases, MUC15 activates the ERK pathway, promoting the progression of glioma, thyroid and pancreatic cancer, leading to poor prognosis 18-20. Additionally, high expression of MUC15 activates the Wnt/β-catenin signaling pathway, supporting invasion and migration in osteosarcoma 21. Conversely, an elevated level of MUC15 has been shown to inhibit metastasis in renal cell carcinoma, suppress tumorigenicity and increase chemoresistance in liver cancer by inhibiting the PI3K/AKT signaling pathway 22-24. Furthermore, aberrant downregulation of MUC15 has been linked to GSK3β phosphorylation, triggering epithelial-mesenchymal transition (EMT) and promoting stemness in prostate cancer 25. MUC15 has garnered increasing attention in the realm of tumor development. However, the expression and effects of MUC15 in NB remain comparatively unknown.

Here, we demonstrated that MUC15 promoted migration of MYCN-NA NB cell lines by suppressing MYCT1/FAK axis. Targeting the metastatic ability of MUC15 in MYCN-NA NB could therefore be a promising and novel therapeutic option.

## 2. Materials and methods

### 2.1 GEO datasets acquisition

The microarray data of NB samples from GSE45547 and GSE49710 were downloaded from the Gene Expression Omnibus (GEO) database. GSE45547 included 608 tumor samples (550 MYCN-NA, 93 MYCN-A, and 6 not applicable), while GSE49710 contained 498 tumor samples (401 MYCN-NA, 92 MYCN-A, and 5 not applicable). The corresponding clinical characteristics for these samples were also acquired.

### 2.2 Construction and validation of mucin-related prognostic signature

We constructed a prognostic gene signature focusing on the mucin family genes (MFGs) within the 401 MYCN-NA samples from the GSE49710 cohort. To minimize the risk of overfitting, we employed LASSO-penalized Cox regression analysis with the 'glmnet' package to create a prognostic model, implementing a 10-fold cross-validation. The independent variable was the normalized expression matrix of MFGs, while the dependent variables were the overall survival (OS) and status of the patients. The penalty parameter (λ) for the model was determined through tenfold cross-validation, selecting the value that resulted in the lowest partial likelihood deviance as the minimum criteria. Then we computed each gene's risk score in this model along with its corresponding regression coefficients, categorized the patients into mucins-high-risk and mucins-low-risk groups based on the median value. The risk score was determined according to the following formula:

score= e^sum (each gene's expression × corresponding coefficient)^

Principal component analysis (PCA) and t-SNE analysis were performed using the "stats" and "Rtsne" packages, respectively. For survival analysis, we established the optimal cut-off expression value using the "survminer" package and evaluated the predictive efficacy of the gene signature via time-dependent ROC curve analyses with the "survivalROC" package.

Utilizing the "limma" package in R, we identified differentially expressed genes (DEGs) between high-risk (83 samples) and low-risk (318 samples) NB groups among the 401 MYCN-NA samples in the GSE49710 cohort. DEGs were selected based on their absolute log2-fold change (|log2FC| > 1) and a false discovery rate (FDR) less than 0.05. The DEGs were visually represented using the "EnhancedVolcano" package in R. Finally, to identify MFGs with prognostic significance, we conducted a univariate Cox regression analysis on the overall survival data, setting a p-value threshold of less than 0.05.

### 2.3 Functional enrichment analysis

For pathway analysis, the Kyoto Encyclopedia of Genes and Genomes (KEGG) and Gene Set Enrichment Analysis (GESA) methods were used to explore significant pathways related to differentially expressed proteins.

### 2.4 Cell lines and cell culture

Human neuroblastoma cell lines (SK-N-SH, SK-N-AS, IMR-32) were purchased from American Type Culture Collection (ATCC, Manassas, VA, USA). The SK-N-SH and IMR32 cell lines were cultured in Eagle's minimum essential medium (EMEM) and SK-N-AS was cultured in Roswell Park Memorial Institute (RPMI-1640) medium. All the mediums containing 10% fetal bovine serum (FBS), 1% Pen/Strep and maintained at 37 °C in 5% CO_2_ incubator.

### 2.5 Plasmid transfection

MUC15 short hairpin RNA (shRNA) was applied to knock-down MUC15 in NB cell lines. pCMV3-MUC15 and pCMV3-MYCT1 vectors were used to overexpress MUC15 and MYCT1, respectively. Ctrl was transfected the empty vector (pcDNA3.1) plasmid. Transient transfection was performed with Lipofectamine 3000 kit (Invitrogen, USA). The quantitative real‐time PCR (RT-PCR) assay was used to evaluate the transfection.

### 2.6 RT-PCR

Total RNA was extracted using TRIzol reagent according to the manufacturer's protocol. Each group of RNA was quantified and inverted into cDNAs using Evo M-MLV RT Master Mix (Accurate Biology, China) according to the manufacturer's instruction. The RT-PCR assay (ABI Q6 System, Applied Biosystems) was used to perform the relative expressions of identified genes.

### 2.7 Transwell assay

2-4×10^4^ tumor cells were harvested and supplemented with 200µl serum-free RIPA-1640 into upper chamber of transwell insert (24 well 8.0-µm pore size, BD Falcon, USA). The lower chamber was added 600µl medium with 10% FBS. Following incubation for 24 h, all the transwell apparatus were fixed with carbinol for 10 min and subsequently stained with 0.1% crystal violet for 15 min at room temperature (RT). Then, the cell count was performed in five randomly selected fields under a light microscope at a magnification of 100×.

### 2.8 Western blot analysis

Cell lysates were obtained by treating with lysis buffer (Thermo Scientific, USA) supplemented with phosphatase inhibitor (MedChemExpress, USA) and protease inhibitor cocktail (Abcam, MA) for 10 min on ice, and centrifuged at 12,000 g for 15 min at 4°C. The resulting supernatants were added into 5× loading buffer and boiled at 95°C for 10 min. For western blot assay, equal concentration of samples was separated by 10% sodium dodecyl sulphate-polyacrylamide gel electrophoresis (SDS/PAGE), then transferred onto 0.45µm Immobilon-P PVDF membranes (Merck Millipore, Ireland) and blocked in western blocking buffer (Beyotime Biotechnology, China) for 2h at RT. After incubating primary antibodies (MUC15, Aanta Cruz Biotechnology, USA; MYCT1 and β-actin, Proteintech, USA, FAK Antibody Sample Kit, Cell Signaling Technology, USA) at 4 °C overnight, the membranes were washed for 3 times with TBST buffer. Subsequently, the membranes were incubated with HRP-conjugated anti-mouse/rabbit secondary antibodies (dilution of 1:20000) and the protein bands were visualized using WB ultrasensitive ECL luminescent liquid (Invigentech, USA).

### 2.9 Cell counting kit-8 (CCK8) assay

The tumor cells were seeded in 96-well cell culture plates. Following transfection as indicated, CCK-8 (Invigentech, USA) was added and incubating at 37°C for 2h. Finally, absorbance was recorded at 450 nm using an enzyme-labeled instrument.

### 2.10 Immunofluorescence staining

Paraffin sections of human NB tissues were subjected to dewaxing and repairing with EDTA antigen retrieval solution (PH=8.0, ZSGB-BIO, China). Blocking was performed using goat serum. The sections were then incubated overnight at 4°C with anti-MUC15 antibody (dilution of 1:100, Santa Cruz Biotechnology, USA). After removing catalase with 0.3% hydrogen peroxide, the sections were labeled with Alexa Fluor 555 Conjugate anti-mouse IgG secondary antibodies (Cell Signaling Technology, USA). Eventually, sections were sealed and nuclei stained by antifade mounting medium containing DAPI (Beyotime Biotechnology, China). Fluorescence images were captured using a Leica TCS SP8 confocal laser scanning microscope.

### 2.11 Cytoskeletal staining

For immunofluorescence imaging, cells were seeded in confocal plates prior to transfection. After treatment, the cells were fixed in 4% paraformaldehyde for 30 minutes. Subsequently, the cells were permeabilized with 0.1% Triton X-100 for 10 minutes. To stain F-actin filaments beneath the cell membranes, FITC-labeled phalloidin (dilution of 1:200, Yeasen Biotechnology, China) was applied and incubated for 90 minutes at RT protected from light The nucleus was stained by antifade mounting medium with DAPI (Beyotime Biotechnology, China). Cell morphology in each group was observed using a fluorescence inverted microscope (Leica DMi8).

### 2.12 Statistical analysis

All the western blot bands presented in this study are representative images obtained from two independent biological replicates. For immunofluorescence, ten images of each stage were randomly selected for statistics. For transwell assay, each group was randomly photographed in five different locations for statistics. Pearson's correlation analysis was employed to calculate the correlations between identified genes. The bar graphs display the means ± standard deviations (SDs). All the data were derived from a minimum of three independent experiments and analyzed using Student's t-test (unpaired, two-tailed) or one-way ANOVA with GraphPad Prism 9.5.1 software (GraphPad, La Jolla, CA, USA). A p-value of less than 0.05 was considered statistically significant.

## Results

### 3.1 Establishment and Validation of a prognostic gene model targeting mucin family

**Figure 1:**
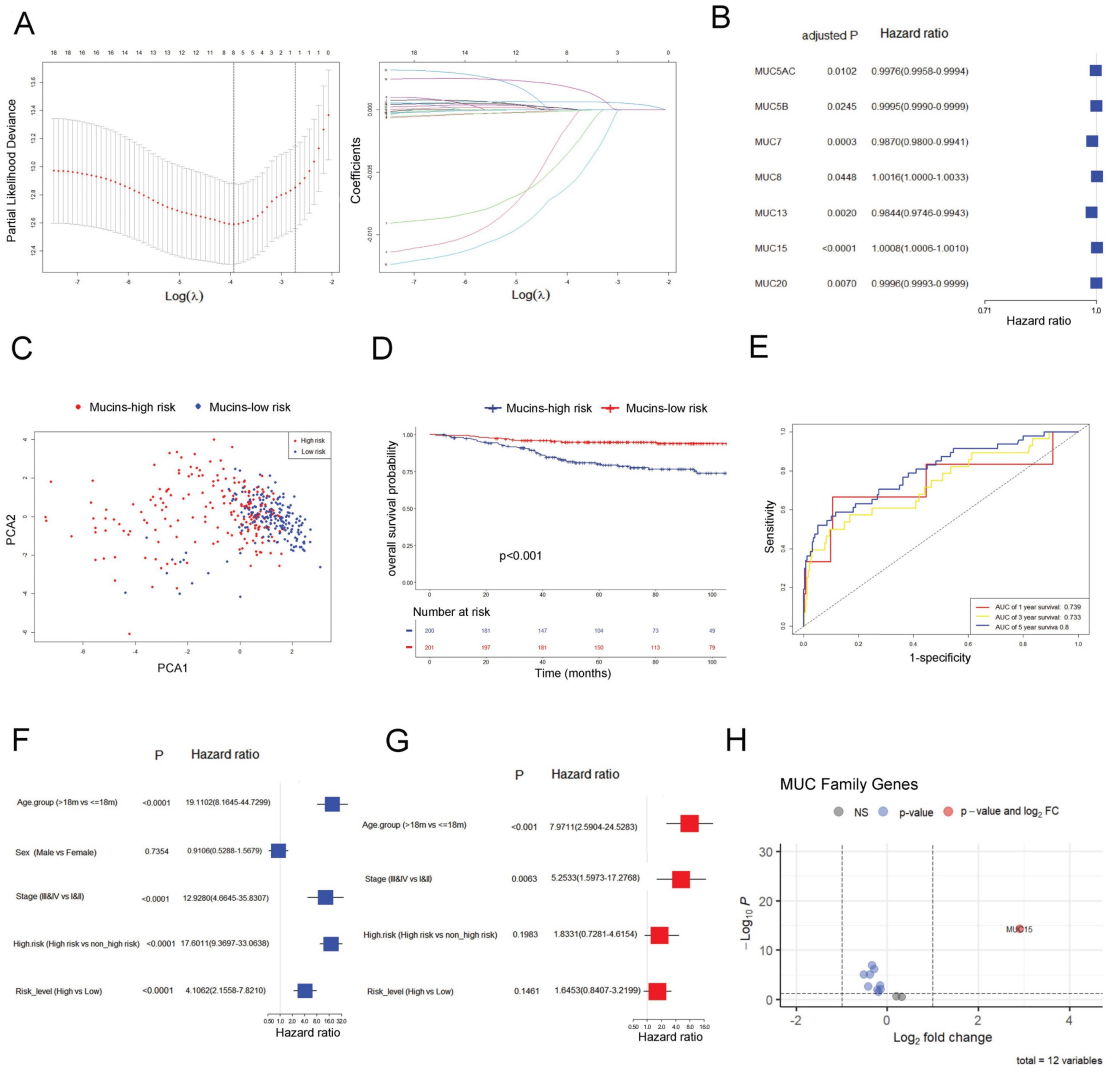
** Establishment and validation of a prognostic gene signature targeting mucin family base on LASSO Cox regression model in GSE49710. A.** Left: Ten times cross-validation for tuning parameter selection in the LASSO model. Right: The LASSO coefficient profiles for clinicopathologic variables. **B.** Forest plots showed the results of the univariate Cox regression analysis of the relationship between gene expression and OS. **C.** PCA plot of mucin-high-risk and mucin-low-risk group in GSE49710. **D.** Kaplan-Meier curves were generated for the OS of patients in the mucin-high-risk and mucin-low-risk groups in GSE49710.** E.** AUC of time-dependent ROC curves were verified the prognostic performance of the risk score in GSE49710. **F-G.** Results of the univariate and multivariate Cox regression analyses regarding OS in the GSE49710 cohort. **H.** Volcano plot visualizing the differentially expressed mucin genes between high-risk and low-risk NB groups in GSE49710.

In GSE49710 dataset, we took advantage of the LASSO algorithm's variable selection and shrinkage properties, to establish an 8-mucin signature model including *MUC12, MUC13, MUC15, MUC20, MUC3A, MUC5AC, MUC7, and MUC8* (Figure 1A). The correlation network of mucin family genes was shown in Figure S1A. Meanwhile, seven of the 8-mucin signature model were significantly associated with the OS of NB patients, especially for *MUC15* (Figure 1B). We subsequently stratified the patients into mucins-high-risk and mucins-low-risk groups based on the median risk score. The distribution of two risk groups was well clustered and discernible (Figure 1C, Figure S1B). Additionally, the risk and status of patients also showed reasonable clustering by mucins-high-risk and mucins-low-risk groups (Figure S1C, D). Kaplan-Meier survival analysis demonstrated that NB patients in the mucins-low-risk group had a superior prognosis (Figure 1D). To validate the prognostic accuracy of our model, we created a time-dependent ROC curve, which demonstrated its ability to accurate predict 1-, 3-, and 5-year OS rates (Figure 1E).

Notably, Cox univariate regression and multivariate Cox regression analyses demonstrated that our 8-mucin signature model could act as an independent prognostic factor for predicting the risk, stage, and age of NB patients (Figure 1F, G). Furthermore, we identified differentially expressed mucins between high-risk and low-risk NB groups, with *MUC15* markedly upregulated in the high-risk cohort (Figure 1H, Figure S1E). The above data revealed that *MUC15* emerged as a unique intersection, underscoring its significant role in the prognostic model.

### 3.2 MUC15 is identified as a potential oncogene in MYCN-NA NB

**Figure 2:**
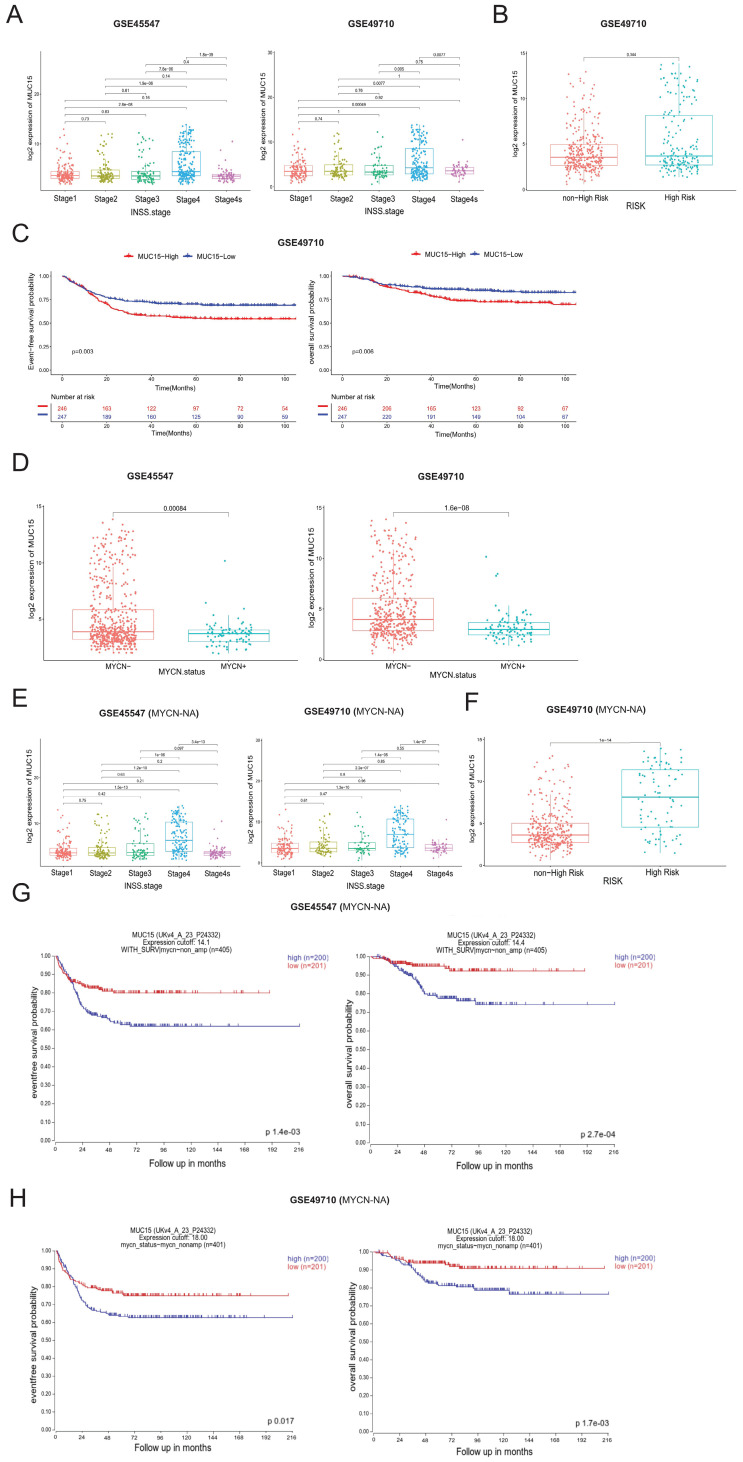
** MUC15 is associated with poor prognosis in MYCN-NA NB from GEO datasets. A.** The expression level of MUC15 across different INSS stages of NB (GSE45547 and GSE49710). **B.** Comparison of MUC15 expression between high-risk and non-high-risk NB cohorts (GSE49710). **C.** Differences in EFS and OS were evaluated in NB samples from GSE49710. **D.** Analysis of MUC15 expression in MYCN-amplified (MYCN-A) and MYCN-non-amplified (MYCN-NA) NB samples (GSE45547 and GSE49710). **E.** The expression of MUC15 across different INSS stages of MYCN-NA NB cohorts (GSE45547 and GSE49710). **F.** Comparison of MUC15 expression between high-risk and non-high-risk MYCN-NA NB cohorts (GSE49710). **G-H.** The EFS and OS of MUC15 in MYCN-NA group of GSE45547 and GSE49710.

Transcriptome analyses of online GEO datasets (GSE45547 and GSE49710) screened out highly expressed MUC15 in advanced NB (INSS Ⅳ stage) (Figure 2A). We were divided into MUC15-high and MUC15-low groups based on the median count of MUC15. In the subset of GSE49710, MUC15 preferred to exist in high-risk patients (Figure 2B) and led to shorter EFS and OS (Figure 2C). Remarkably, MUC15 was obvious upregulated in MYCN-NA cohort compared with MYCN-A (Figure 2D). The expression difference in advanced stage and high-risk of NB was more significant under MYCN-NA cohort (Figure 2E, F). In particular, increased MUC15 was more related to the shorter EFS and OS in MYCN-NA cohort (Figure 2G, H), indicating *MUC15* may serve as a potential oncogene to predict prognosis in MYCN-NA patients. Together, these observations demonstrated that *MUC15* is involved in carcinogenesis of MYCN-NA NB.

### 3.3 MUC15 is highly expressed in MYCN-NA NB

**Figure 3:**
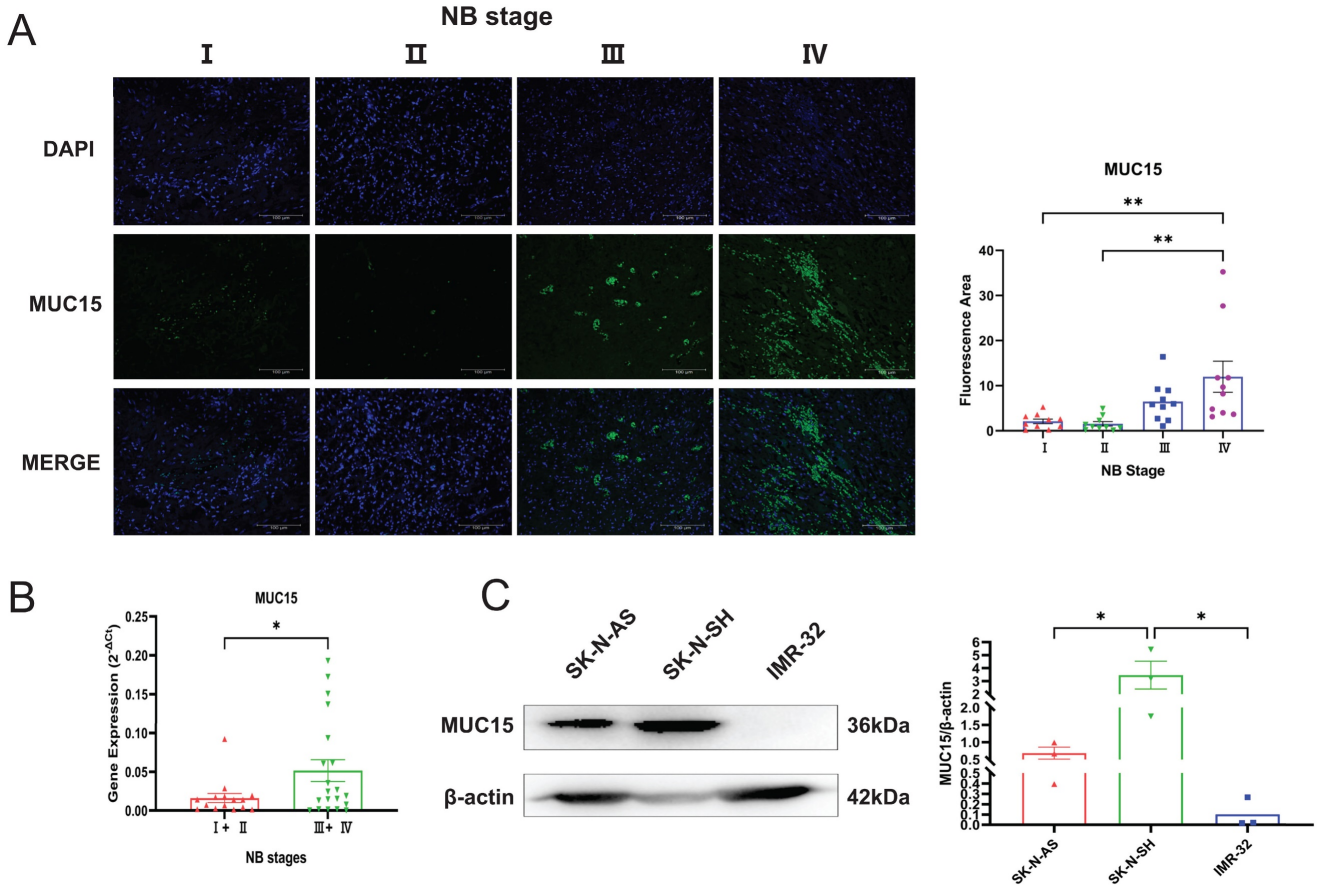
**MUC15 is highly expressed in MYCN-NA NB. A.** Expression of MUC15 in tissues with different stages. Blue and green fluorescence represent DAPI (nucleus) and MUC15, respectively. The histogram showed the relative fluorescence area (n=10). **B.** RT-PCR showed the MUC15 expression in patients with different stages (I+II, n=15; Ⅲ+Ⅳ, n=20). **C.** Western blotting revealed the ratio of MUC15 to internal reference protein in three different neuroblastoma cell lines. *p < 0.05, **p < 0.01.

We performed immunofluorescence staining and RT-PCR assay to investigate the express situation of MUC15 in MYCN-NA NB slices. As was shown in Figure 3A and B, MUC15 was enriched in Ⅲ, Ⅳ stages compared to Ⅰ, Ⅱ stages. Interestingly, we observed MUC15 content in IMR-32 (MYCN-A cell line) had lower expression than SK-N-SH and SK-N-AS (MYCN-NA cell lines) (Figure 3C). Thus, we confirmed that MUC15 is highly expressed in MYCN-NA NB with poor prognosis.

### 3.4 MUC15 promotes NB cell migration in *vitro*

In order to elucidate the mechanism of MUC15, NB cells subjected to MUC15 knocking-down or full-length overexpression were first validated by RT-PCR and immunoblotting (Figure S2A, B). We then carried out whether MUC15 influence the proliferation and chemoresistance of NB. As shown in Figure S2C and D*,* endogenous MUC15 alteration or exogeneous MUC15 exerted trivial effects on proliferation of SK-N-AS and SK-N-SH cells with or without cisplatin treatment.

**Figure 4:**
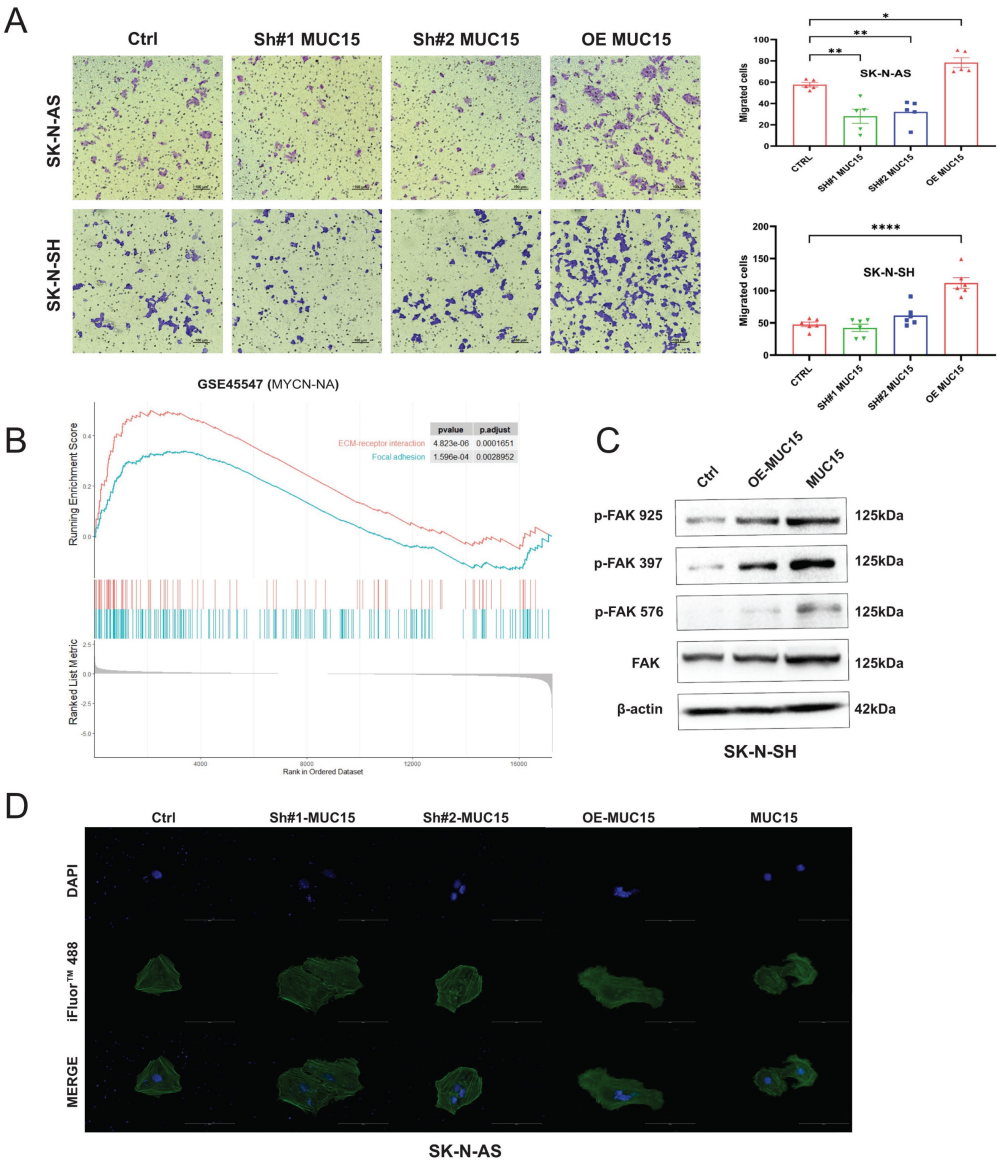
** MUC15 promotes NB cell migration. A.** Left: Transwell images of migration for NB cells randomly selected from each treatment group. Right: The bar chart showed the statistics of migration results of SK-N-AS and SK-N-SH. **B.** GESA analysis of migration-related pathways in the MYCN-NA subgroup of GSE45547.** C.** Western blotting showed increased expressions of three phosphorylated FAKs exposing to MUC15 overexpression and exogeneous MUC15 (20ng/ml) in SK-N-SH. **D.** Cytoskeletal protein staining in SK-N-AS. The green fluorescence for phalloidin combined with filamentous actin (F-actin) and excited with 488nm photoexcitation. The blue staining is DAPI for nuclear staining and MERGE as an overlapping image of two channels. SH#1&2 MUC15, knockdown of MUC15; OE-MUC15, overexpression of MUC15; MUC15, exogeneous MUC15 protein (20ng/ml). *p < 0.05, **p < 0.01, ****P<0.0001.

Considering migration-related pathways such as focal adhesion and ECM-receptor interaction were positive enriched in GSE45447 set (Figure S2E), migration abilities were further explored. We noticed that overexpression MUC15 vastly increased the migration of both SK-N-AS and SK-N-SH cells (Figure 4A). Knocking-down MUC15 decreased the migration of SK-N-AS, but seldom affected SK-N-SH. In addition, the enrichment of ECM-receptor interaction and focal adhesion pathway in MYCN-NA cohort supported the conclusion that migration-related genes were highly expressed (Figure 4B). Furthermore, western blotting results suggested that the level of p-FAK (925), p-FAK (397) and p-FAK (576) were increased under MUC15 overexpression and exogeneous MUC15 in SK-N-SH (Figure 4C). Cytoskeletal fluorescence staining in SK-N-AS showed increased and irregular skeletal proteins in MUC15 overexpression and exogeneous MUC15 groups, the appearance of filopodia indicated enhanced migration ability of above two groups (Figure 4D). To sum up, overexpression MUC15 promoted migration of MYCN-NA NB cells in vitro.

### 3.5 MUC15 facilitates migration via MYCT1

**Figure 5:**
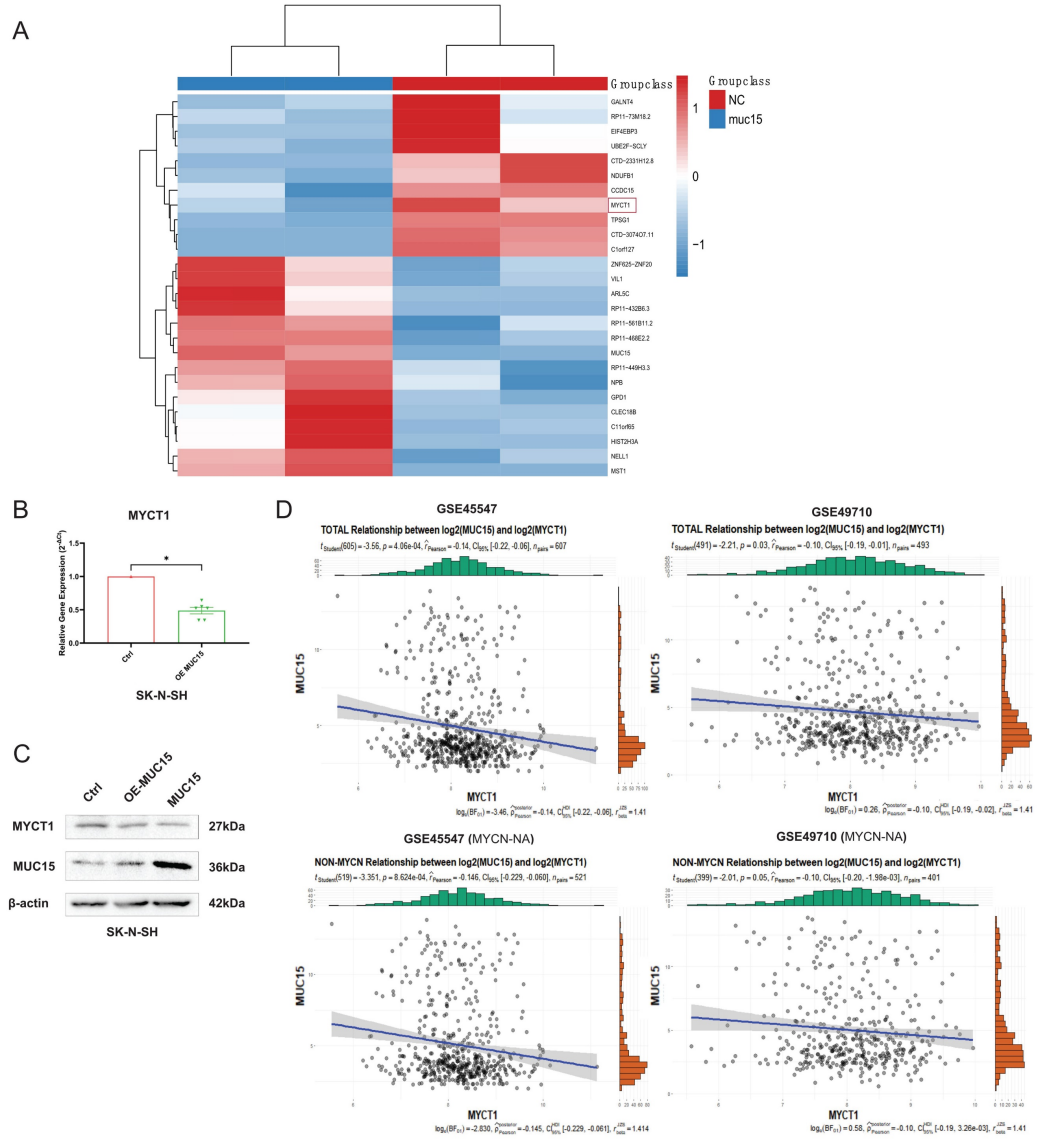
** MUC15 facilitates migration via MYCT1. A.** Transcriptome analysis of MUC15 overexpression and control groups. **B.** MYCT1 expression situation after overexpression of MUC15 by RT-PCR in SK-N-SH (n=6). **C.** The content of MYCT1 after MUC15 overexpression in SK-N-SH was detected by western blotting. **D.** The correlation analysis between MUC15 and MYCT1 in GSE45547 and GSE49710. OE-MUC15, overexpression of MUC15; MUC15, exogeneous MUC15 protein (20ng/ml). *p < 0.05.

To figure out the downstream mechanism of MUC15, we compared the RNA sequences of overexpressed MUC15 and control groups in IMR-32. As shown in Figure 5A, we found MYCT1 reduced after overexpressing MUC15. RT-PCR as well as western blotting of SK-N-SH also validated the consequence (Figure 5B, C). Furthermore, correlation analysis revealed that MYCT1 was negatively associated with MUC15, whether in total samples or MYCN-NA subgroups (GSE45547 and GSE49710) (Figure 5D).

### 3.6 MYCT1 rescues the MUC15-induced migration of NB

**Figure 6:**
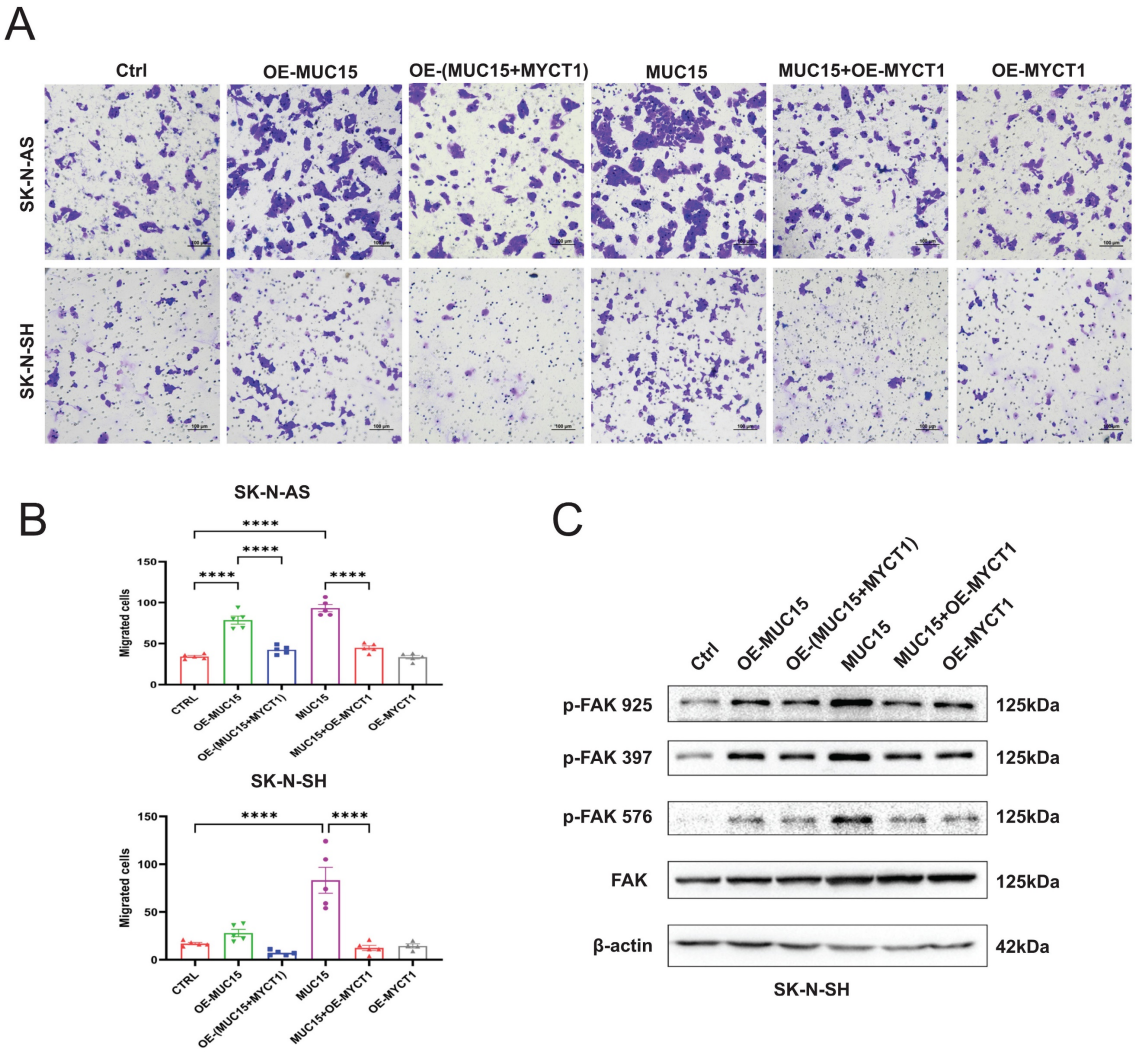
**MYCT1 rescues the MUC15-induced migration of NB. A.** Transwell images of migration for NB cells were randomly selected from each group treated as indicated. **B.** The statistics analysis of SK-N-AS and SK-N-SH in A. **C.** Western blotting showed the expressions of three FAK phosphorylation in SK-N-SH treated as indicated. OE-MUC15, overexpression of MUC15; OE-(MUC15+MYCT1), overexpression of MUC15 and MYCT1; MUC15, exogeneous MUC15 protein (20ng/ml); MUC15+OE-MYCT1, exogeneous MUC15 protein (20ng/ml) and overexpression of MYCT1; OE-MYCT1, overexpression of MYCT1. ****P<0.0001.

Later, the full length MYCT1 plasmid was constructed and transfected into MUC15-overexpressing or exogeneous MUC15-treated cell lines. Our results showed that MUC15-induced migration was depressed by MYCT1 in both overexpression and exogeneous MUC15-treated groups among SK-N-AS and SK-N-SH (Figure 6A, B). At the same time, western blotting in SK-N-SH also showed that phosphorylation of FAK were all inhibited, especially in the exogeneous MUC15-treated subgroup (Figure 6C), indicating that the phosphorylation of FAK induced by MUC15 could be inhibited by MYCT1. Totally, our study demonstrated MUC15 as an independent prognostic factor to predict the prognosis of NB patients with MYCN-NA. MYCT1 acted as an inhibitory factor in the process of MUC15 promoting migration of NB.

## 4. Discussion

Investigating the role of membrane proteins in neuroblastoma is critical in tackling the challenge of discovering novel drug targets and precisely targeting the microenvironment. Transmembrane mucins expressed in tumors play a crucial role in promoting proliferation, metastasis, and chemoresistance. In this study, we conducted a comprehensive analysis of expression matrix of mucin family genes using the online GEO database (GSE49710). From this analysis, we developed an 8-mucin signature model that specifically targeted mucin family genes. By categorizing NB patients into mucins-low-risk and mucins-high-risk groups based on this model, we were able to demonstrate that the model had superior prognostic predictive capabilities for NB patients, including risk, stage and age. Notably, we observed significant upregulation of MUC15 in the high-risk NB group.

The extracellular domain of MUC15 contains N- and O-glycosylated modifications, alterations in N-glycan had been shown to decrease FAK phosphorylation and were associated with tumor migration 26. FAK, the intersection of multiple signaling pathways for tumors, is a crucial tyrosine kinase protein that regulates cell migration and adhesion. FAK exists in an inactive form in the cytoplasm, but after phosphorylation activation, it interacts with membrane receptors and nucleus transcription factors to transmit diverse signals 27. FAK promotes tumor development. In fact, several FAK phosphorylation inhibitors have entered clinical trials, highlighting the therapeutic potential of targeting this protein in cancer treatment 28.

The MYC family, which includes c-Myc, N-Myc and L-Myc transcription factors, plays a crucial role in tumorigenesis, maintenance and progression in various cancers. Each subtype of MYC exhibits unique features, leading to distinct oncogenic mechanisms in tumor development 29. In NB, N-Myc (MYCN) amplification is a common variation occurring in approximately 25% of cases, which contributes to malignant proliferation and metastasis 30. With the deepening of research, the mechanism of c-Myc affects NB has attracted increasing attention in recent years 31-34. However, the exact mechanism of c-Myc in NB remains largely unclear.

As a direct downstream target of c-Myc, MYCT1 has two E-boxes in front of its promoter, which can bind c-Myc transcription complex 35, 36. MYCT1, also known as MT-MC1 or MTCL, was initially discovered in myeloid cells 37. MYCT1 plays inhibitory roles in controlling the growth, adhesion, transformation, apoptosis and migration in various cancers 38-43. However, not all evidence supports MYCT1 as a tumor suppressor gene. Kabir et al. reported that MYCT1 regulated actin cytoskeleton in endothelial motility to promote tumor angiogenesis. Targeting MYCT1 enhanced antitumor immunity in the microenvironment and improved the effectiveness of anti-PD1 treatment 44. Based on our transcriptome data and mechanistic work, we have identified MYCT1 as a factor that dampened MUC15-induced FAK phosphorylation and NB cell migration, which is consistent with its migration-inhibiting activity.

In summary, we have discovered that MUC15 served as a potential target and novel biomarker for NB prognosis assessment. Overexpression of MUC15 significantly enhanced NB cell migration. Mechanically, MYCT1 served as a negative regulator of elevated MUC15, thereby reducing the MUC15-induced migration of NB cells. Further investigation is needed to clarify the underlying mechanism of how MUC15 influences MYCT1 and to establish MUC15 as a candidate for assessing prognosis in NB patients with MYCN-NA. Our study is the first to identify molecular marker and signaling pathway for MYCN-NA NB membrane surface protein. Our findings complemented the understanding of NB pathogenesis, providing a foundation for future precision treatments.

## Supplementary Material

Supplementary figures.

## Abbreviations

EFS: event-free survival
FAK: focal adhesion kinase
MYCN-A: MYCN amplificated
MYCN-NA: MYCN non-amplificated
MUC: mucin
MYCT1: MYC target 1
NB: neuroblastoma
OS: overall survival
