# Supplementary material for: MUC15 is an independent prognostic factor that promotes metastases of MYCN non-amplified neuroblastoma
Source: J Cancer. 2023 Oct 16;14(18):3496–507. doi: 10.7150/jca.89360 (PMC10647185; doi:10.7150/jca.89360)
Supplement: Supplementary file 1 — Supplementary figures. [file jcav14p3496s1.pdf]

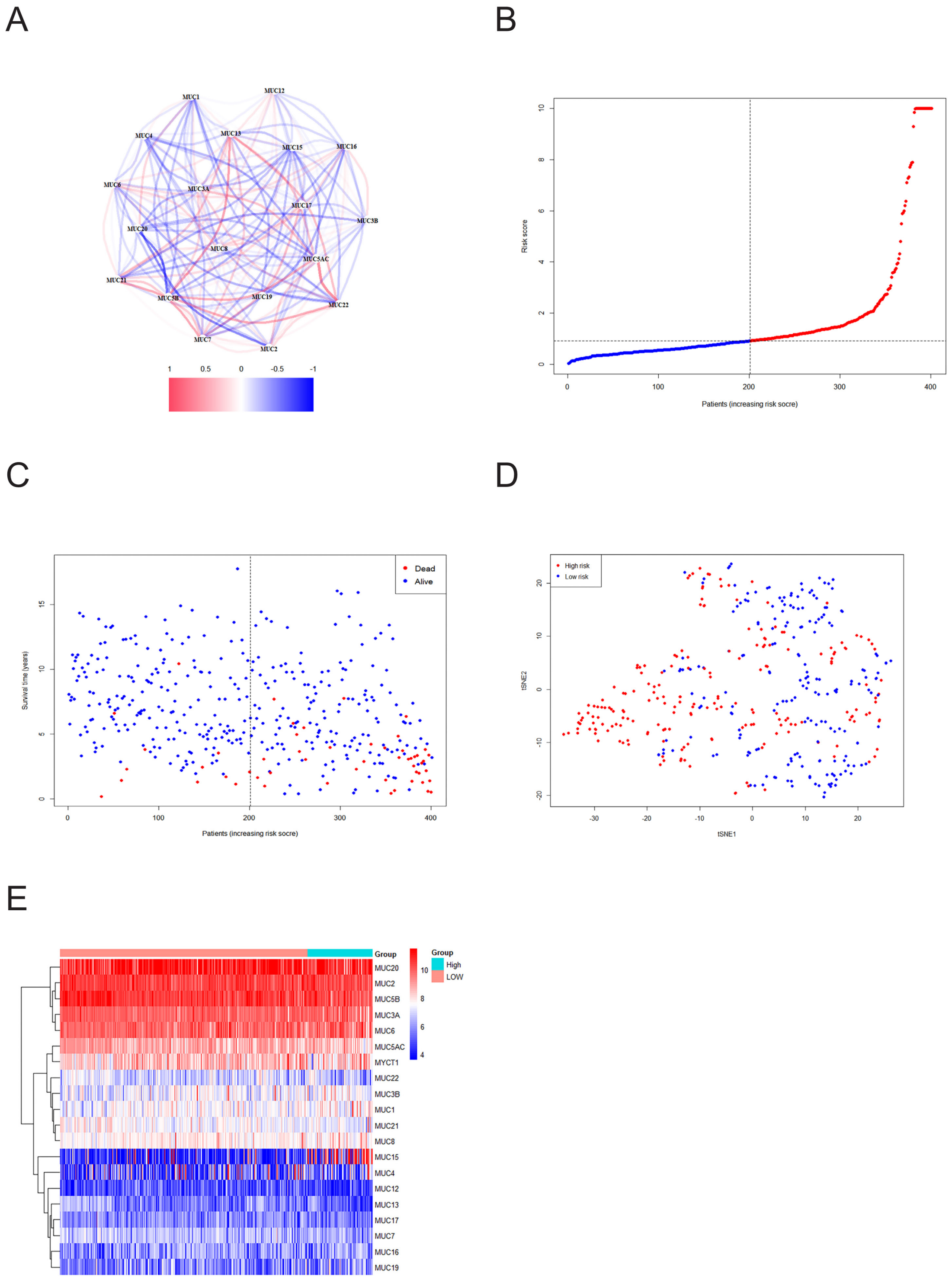

Supplementary Figure 1. The prognostic prediction of the 8-mucins signature model. A. The correlation network of mucin family genes. The correlation coefficients were represented by different colors. **B-D** The

distribution and median value of the risk scores, as well as t-SNE analysis in the GSE49710. **E.** Heatmap of expression of 19 genes for mucin family genes.

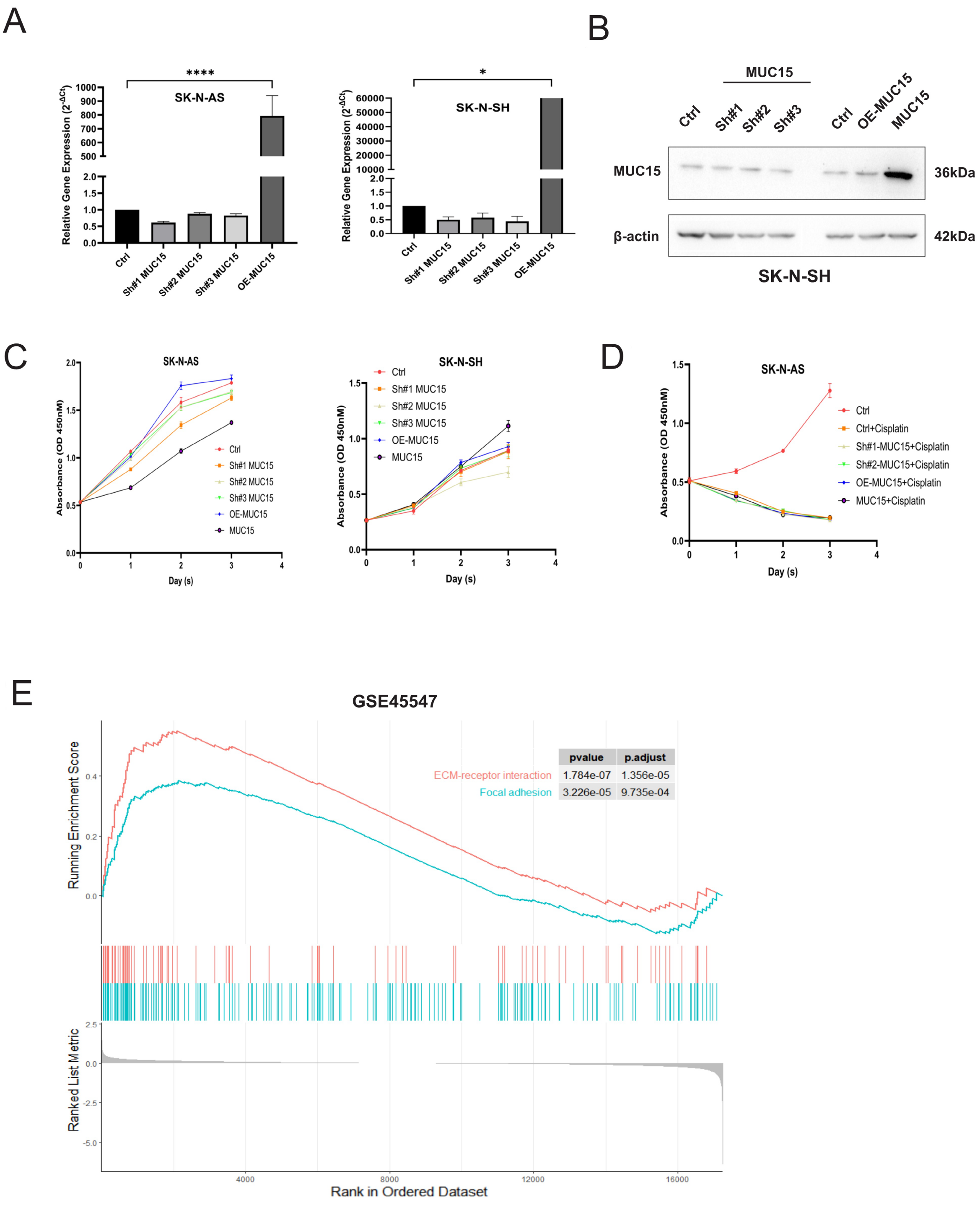

Supplementary Figure 2. The effects of MUC15 on NB cell lines. A. RT-PCR results of knockdown and overexpression of MUC15 in NB cell lines. **B.** Western blotting results of knockdown and overexpression of MUC15. **C-D.**

Effects of knockdown and overexpression of MUC15 on cell proliferation and chemoresistance. **E.** GESA analysis of migration-related pathways in GSE45547
